# Bis{2,4-dibromo-6-[(*E*)-(4-fluoro­benz­yl)imino­meth­yl]phenolato-κ^2^ *N*,*O*}cobalt(II)

**DOI:** 10.1107/S1600536812043929

**Published:** 2012-10-31

**Authors:** Hong Yu, Zhu-Jun Chen, Yue-Bao Jin, Yong-Kang Chang, Ke-Wei Lei

**Affiliations:** a State Key Lab. Base of Novel Functional Materials and Preparation Science, Institute of Solid Materials Chemistry, Faculty of Materials Science and Chemical Engineering, Ningbo University, Ningbo 315211, People’s Republic of China; b Zhejiang Textile and Fashion College, Ningbo 315211, People?s Republic of China

## Abstract

The complete mol­ecule of the title complex, [Co(C_14_H_9_Br_2_FNO)_2_], is generated by crystallographic twofold symmetry, with the Co^II^ atom lying on the rotation axis. The coordination of the metal atom by the two *N*,*O*-bidentate ligands results in a squashed CoN_2_O_2_ tetra­hedron. The six-membered chelate ring is an envelope, with the metal atom as the flap. The dihedral angle between the planes of the aromatic rings within each ligand is 84.1 (6)°.

## Related literature

For a related structure, see: Jadeja & Shah (2007 ▶). 
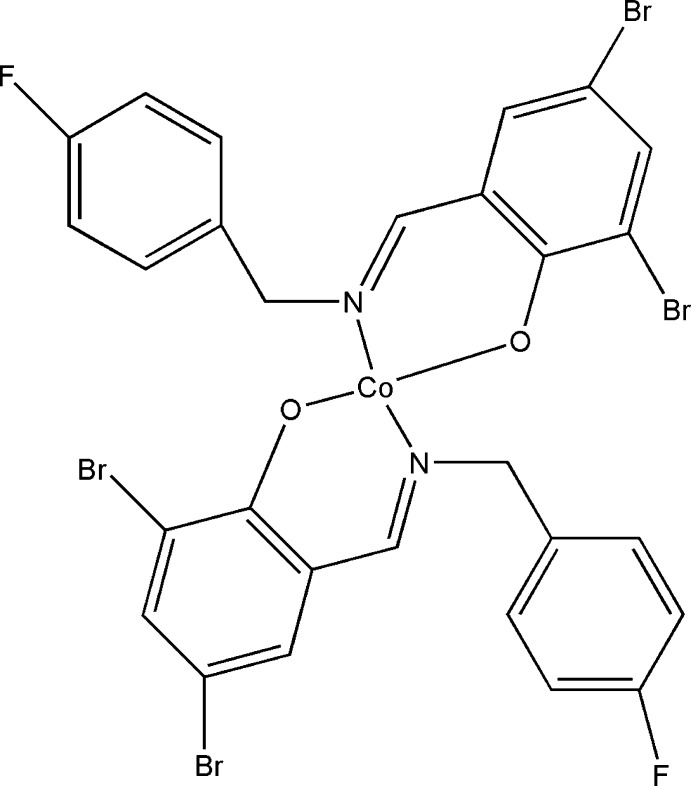


## Experimental

### 

#### Crystal data

[Co(C_14_H_9_Br_2_FNO)_2_]
*M* *_r_* = 830.97
Monoclinic, 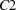

*a* = 14.6921 (14) Å
*b* = 9.7598 (3) Å
*c* = 13.1195 (13) Å
β = 133.608 (17)°
*V* = 1362.2 (4) Å^3^
*Z* = 2
Mo *K*α radiation
μ = 6.54 mm^−1^
*T* = 293 K
0.33 × 0.21 × 0.12 mm

#### Data collection

Rigaku R-AXIS RAPID CCD diffractometer
Absorption correction: multi-scan (*ABSCOR*; Higashi, 1995 ▶) *T*_min_ = 0.210, *T*_max_ = 0.456
5582 measured reflections
2437 independent reflections
2161 reflections with *I* > 2σ(*I*)
*R*_int_ = 0.035

#### Refinement

*R*[*F* ^2^ > 2σ(*F* ^2^)] = 0.037
*wR*(*F* ^2^) = 0.079
*S* = 1.02
2437 reflections
177 parameters
1 restraint
H-atom parameters constrained
Δρ_max_ = 0.44 e Å^−3^
Δρ_min_ = −0.46 e Å^−3^
Absolute structure: Flack (1983 ▶), 961 Friedel pairs
Flack parameter: −0.012 (14)

### 

Data collection: *RAPID-AUTO* (Rigaku, 1998 ▶); cell refinement: *RAPID-AUTO*; data reduction: *CrystalStructure* (Rigaku/MSC, 2004 ▶); program(s) used to solve structure: *SHELXS97* (Sheldrick, 2008 ▶); program(s) used to refine structure: *SHELXL97* (Sheldrick, 2008 ▶); molecular graphics: *SHELXTL* (Sheldrick, 2008 ▶); software used to prepare material for publication: *SHELXL97*.

## Supplementary Material

Crystal structure: contains datablock(s) global, I. DOI: 10.1107/S1600536812043929/hb6941sup1.cif

Structure factors: contains datablock(s) I. DOI: 10.1107/S1600536812043929/hb6941Isup2.hkl

Additional supplementary materials:  crystallographic information; 3D view; checkCIF report

## Figures and Tables

**Table:** 

Co1—O1	1.933 (4)
Co1—N1	2.023 (5)

**Table:** 

O1—Co1—O1^i^	143.6 (2)
O1—Co1—N1	92.90 (17)
O1^i^—Co1—N1	104.12 (18)
N1—Co1—N1^i^	123.7 (3)

Symmetry code: (i) 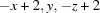.

**Fig. 1.**
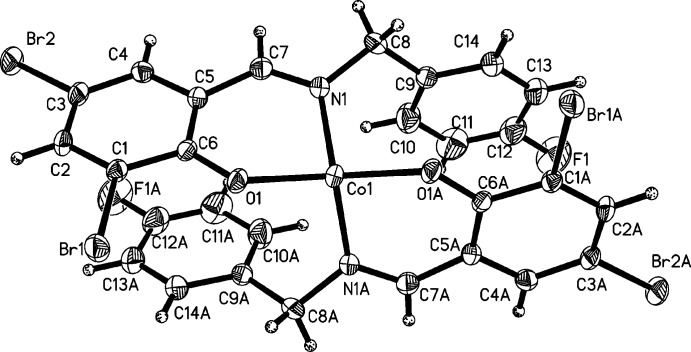
The molecular structure of the title complex, showing 30% probability displacement ellipsoids. Atoms with label suffix A are generated by (2–x, –y, 2–z).

## supplementary crystallographic information

### Experimental

Synthesis of the ligand 2-((*E*)-(4-fluorobenzylimino) methyl)-4,6-dibromophenol: 3,5-dibromo-2-hydroxybenzal dehyde and (4-fluorophenyl)methanamine (1:1) were dissolved in ethanol and the solution was refluxed for 2 h. After evaporation, a crude product was recrystallized twice from ethanol solution to give a yellow product.

1 mmol (0.77 g) of the ligand and 0.5 mmol (0.145 g) Co(NO_3_)_2_ were dissolved in ethanol and the solutions mixed and stirred for about 10 minutes. The slow vaporization of the solvent yielded after about 3 d dark red single blocks. Yield: 72.3%. Calcd. for C_28_H_18_Br_4_CoF_2_N_2_O_2_: C 40.37; H 2.42; O 3.84; N 3.36; Found: C 40.26; H 2.40; O 3.83; N 3.35%.

### Refinement

All H atoms were placed in geometrically idealized positions and constrained to ride on their parent atoms (C—H = 0.93%A) and *U*_iso_(H) values equal to 1.2 *U*_eq_(C).

### Crystal data

**Table:**

[Co(C_14_H_9_Br_2_FNO)_2_]	*Z* = 2
*M**_r_* = 830.97	*F*(000) = 802
Monoclinic, *C*2	*D*_x_ = 2.026 Mg m^−^^3^
Hall symbol: C 2y	Mo *K*α radiation, λ = 0.71073 Å
*a* = 14.6921 (14) Å	θ = 2.8–26.4°
*b* = 9.7598 (3) Å	µ = 6.54 mm^−^^1^
*c* = 13.1195 (13) Å	*T* = 293 K
β = 133.608 (17)°	Block, red
*V* = 1362.2 (4) Å^3^	0.33 × 0.21 × 0.12 mm

### Data collection

**Table:**

Rigaku R-AXIS RAPID CCD diffractometer	2437 independent reflections
Radiation source: fine-focus sealed tube	2161 reflections with *I* > 2σ(*I*)
Graphite monochromator	*R*_int_ = 0.035
ω scans	θ_max_ = 26.4°, θ_min_ = 2.8°
Absorption correction: multi-scan (*ABSCOR*; Higashi, 1995)	*h* = −16→18
*T*_min_ = 0.210, *T*_max_ = 0.456	*k* = −12→11
5582 measured reflections	*l* = −16→16

### Refinement

**Table:**

Refinement on *F*^2^	Secondary atom site location: difference Fourier map
Least-squares matrix: full	Hydrogen site location: inferred from neighbouring sites
*R*[*F*^2^ > 2σ(*F*^2^)] = 0.037	H-atom parameters constrained
*wR*(*F*^2^) = 0.079	*w* = 1/[σ^2^(*F*_o_^2^) + (0.0352*P*)^2^] where *P* = (*F*_o_^2^ + 2*F*_c_^2^)/3
*S* = 1.02	(Δ/σ)_max_ = 0.001
2437 reflections	Δρ_max_ = 0.44 e Å^−^^3^
177 parameters	Δρ_min_ = −0.46 e Å^−^^3^
1 restraint	Absolute structure: Flack (1983), 961 Friedel pairs
Primary atom site location: structure-invariant direct methods	Flack parameter: −0.012 (14)

### Special details

**Table:**

Geometry. All e.s.d.'s (except the e.s.d. in the dihedral angle between two l.s. planes) are estimated using the full covariance matrix. The cell e.s.d.'s are taken into account individually in the estimation of e.s.d.'s in distances, angles and torsion angles; correlations between e.s.d.'s in cell parameters are only used when they are defined by crystal symmetry. An approximate (isotropic) treatment of cell e.s.d.'s is used for estimating e.s.d.'s involving l.s. planes.
Refinement. Refinement of *F*^2^ against ALL reflections. The weighted *R*-factor *wR* and goodness of fit *S* are based on *F*^2^, conventional *R*-factors *R* are based on *F*, with *F* set to zero for negative *F*^2^. The threshold expression of *F*^2^ > σ(*F*^2^) is used only for calculating *R*-factors(gt) *etc*. and is not relevant to the choice of reflections for refinement. *R*-factors based on *F*^2^ are statistically about twice as large as those based on *F*, and *R*- factors based on ALL data will be even larger.

### Fractional atomic coordinates and isotropic or equivalent isotropic displacement parameters (Å^2^)

**Table:**

	*x*	*y*	*z*	*U*_iso_*/*U*_eq_	
Br1	1.04553 (6)	0.62756 (5)	1.37969 (7)	0.04722 (19)	
Br2	0.66469 (6)	0.25233 (7)	1.20378 (7)	0.0518 (2)	
Co1	1.0000	0.46517 (11)	1.0000	0.0378 (3)	
F1	1.0441 (5)	0.0042 (6)	0.6661 (7)	0.108 (2)	
O1	0.9762 (3)	0.5270 (4)	1.1203 (4)	0.0377 (9)	
N1	0.8324 (4)	0.3673 (5)	0.8675 (5)	0.0380 (11)	
C1	0.9177 (5)	0.5015 (5)	1.2457 (6)	0.0317 (12)	
C2	0.8477 (5)	0.4400 (6)	1.2682 (6)	0.0354 (13)	
H2	0.8609	0.4636	1.3462	0.043*	
C3	0.7584 (5)	0.3434 (6)	1.1736 (6)	0.0372 (14)	
C4	0.7374 (5)	0.3097 (6)	1.0577 (6)	0.0358 (13)	
H4	0.6756	0.2459	0.9939	0.043*	
C5	0.8088 (5)	0.3712 (6)	1.0336 (6)	0.0323 (12)	
C6	0.9041 (5)	0.4673 (5)	1.1321 (6)	0.0294 (12)	
C7	0.7747 (5)	0.3358 (6)	0.9051 (6)	0.0395 (14)	
H7	0.7020	0.2837	0.8416	0.047*	
C8	0.7686 (5)	0.3365 (7)	0.7212 (6)	0.0437 (15)	
H8A	0.7490	0.4220	0.6718	0.052*	
H8B	0.6898	0.2904	0.6748	0.052*	
C9	0.8460 (5)	0.2477 (7)	0.7099 (6)	0.0373 (13)	
C10	0.9141 (7)	0.1382 (8)	0.7983 (8)	0.0622 (19)	
H10	0.9152	0.1193	0.8686	0.075*	
C11	0.9810 (8)	0.0557 (8)	0.7835 (11)	0.076 (3)	
H11	1.0272	−0.0183	0.8435	0.092*	
C12	0.9781 (7)	0.0843 (8)	0.6809 (9)	0.063 (2)	
C13	0.9111 (6)	0.1905 (8)	0.5902 (8)	0.0566 (18)	
H13	0.9091	0.2069	0.5189	0.068*	
C14	0.8456 (5)	0.2737 (7)	0.6069 (7)	0.0431 (15)	
H14	0.8008	0.3483	0.5474	0.052*	

### Atomic displacement parameters (Å^2^)

**Table:**

	*U*^11^	*U*^22^	*U*^33^	*U*^12^	*U*^13^	*U*^23^
Br1	0.0476 (3)	0.0584 (4)	0.0421 (4)	−0.0195 (3)	0.0334 (3)	−0.0154 (3)
Br2	0.0528 (4)	0.0658 (4)	0.0576 (5)	−0.0160 (3)	0.0460 (4)	−0.0025 (3)
Co1	0.0322 (6)	0.0575 (7)	0.0337 (6)	0.000	0.0265 (5)	0.000
F1	0.113 (4)	0.110 (4)	0.138 (5)	0.045 (4)	0.101 (4)	0.006 (4)
O1	0.035 (2)	0.049 (2)	0.041 (2)	−0.0131 (18)	0.031 (2)	−0.0094 (18)
N1	0.031 (2)	0.056 (3)	0.030 (3)	0.002 (2)	0.022 (2)	−0.001 (2)
C1	0.031 (3)	0.034 (3)	0.035 (3)	−0.008 (2)	0.025 (3)	−0.005 (2)
C2	0.043 (3)	0.040 (3)	0.041 (4)	−0.003 (3)	0.035 (3)	−0.001 (3)
C3	0.036 (3)	0.045 (3)	0.042 (4)	−0.008 (3)	0.032 (3)	−0.002 (3)
C4	0.033 (3)	0.039 (3)	0.033 (3)	−0.005 (3)	0.022 (3)	−0.003 (3)
C5	0.032 (3)	0.038 (3)	0.031 (3)	−0.001 (3)	0.023 (3)	0.002 (2)
C6	0.028 (3)	0.033 (3)	0.032 (3)	0.004 (2)	0.022 (3)	0.006 (2)
C7	0.034 (3)	0.048 (3)	0.036 (4)	−0.009 (3)	0.024 (3)	−0.009 (3)
C8	0.034 (3)	0.063 (4)	0.026 (3)	0.002 (3)	0.018 (3)	−0.003 (3)
C9	0.033 (3)	0.046 (3)	0.033 (3)	−0.010 (3)	0.023 (3)	−0.011 (3)
C10	0.071 (5)	0.066 (5)	0.067 (5)	0.008 (4)	0.055 (4)	0.015 (4)
C11	0.079 (5)	0.059 (5)	0.099 (8)	0.029 (4)	0.065 (6)	0.024 (5)
C12	0.061 (5)	0.069 (5)	0.072 (6)	0.005 (4)	0.051 (5)	−0.011 (4)
C13	0.053 (4)	0.076 (5)	0.050 (4)	−0.002 (4)	0.039 (4)	−0.008 (4)
C14	0.043 (3)	0.049 (4)	0.040 (4)	0.001 (3)	0.030 (3)	−0.002 (3)

### Geometric parameters (Å, º)

**Table:**

Br1—C1	1.891 (5)	C5—C6	1.419 (7)
Br2—C3	1.896 (5)	C5—C7	1.430 (8)
Co1—O1	1.933 (4)	C7—H7	0.9300
Co1—O1^i^	1.933 (4)	C8—C9	1.515 (8)
Co1—N1	2.023 (5)	C8—H8A	0.9700
Co1—N1^i^	2.023 (5)	C8—H8B	0.9700
F1—C12	1.359 (8)	C9—C14	1.371 (8)
O1—C6	1.308 (6)	C9—C10	1.374 (10)
N1—C7	1.279 (7)	C10—C11	1.383 (11)
N1—C8	1.474 (8)	C10—H10	0.9300
C1—C2	1.387 (8)	C11—C12	1.346 (12)
C1—C6	1.400 (7)	C11—H11	0.9300
C2—C3	1.380 (8)	C12—C13	1.358 (10)
C2—H2	0.9300	C13—C14	1.388 (8)
C3—C4	1.367 (8)	C13—H13	0.9300
C4—C5	1.420 (7)	C14—H14	0.9300
C4—H4	0.9300		
			
O1—Co1—O1^i^	143.6 (2)	N1—C7—C5	127.6 (5)
O1—Co1—N1	92.90 (17)	N1—C7—H7	116.2
O1^i^—Co1—N1	104.12 (18)	C5—C7—H7	116.2
O1—Co1—N1^i^	104.12 (18)	N1—C8—C9	113.4 (5)
O1^i^—Co1—N1^i^	92.90 (17)	N1—C8—H8A	108.9
N1—Co1—N1^i^	123.7 (3)	C9—C8—H8A	108.9
C6—O1—Co1	125.1 (3)	N1—C8—H8B	108.9
C7—N1—C8	117.3 (5)	C9—C8—H8B	108.9
C7—N1—Co1	121.3 (4)	H8A—C8—H8B	107.7
C8—N1—Co1	121.3 (4)	C14—C9—C10	118.7 (6)
C2—C1—C6	122.7 (5)	C14—C9—C8	119.8 (6)
C2—C1—Br1	119.3 (4)	C10—C9—C8	121.5 (6)
C6—C1—Br1	117.8 (4)	C9—C10—C11	120.6 (7)
C3—C2—C1	119.3 (5)	C9—C10—H10	119.7
C3—C2—H2	120.4	C11—C10—H10	119.7
C1—C2—H2	120.4	C12—C11—C10	119.0 (7)
C4—C3—C2	120.6 (5)	C12—C11—H11	120.5
C4—C3—Br2	118.7 (4)	C10—C11—H11	120.5
C2—C3—Br2	120.6 (4)	C11—C12—C13	122.6 (7)
C3—C4—C5	120.8 (5)	C11—C12—F1	119.4 (8)
C3—C4—H4	119.6	C13—C12—F1	118.1 (8)
C5—C4—H4	119.6	C12—C13—C14	118.1 (7)
C6—C5—C4	119.5 (5)	C12—C13—H13	121.0
C6—C5—C7	123.8 (5)	C14—C13—H13	121.0
C4—C5—C7	116.6 (5)	C9—C14—C13	121.1 (6)
O1—C6—C1	119.1 (5)	C9—C14—H14	119.5
O1—C6—C5	123.9 (5)	C13—C14—H14	119.5
C1—C6—C5	117.0 (5)		
			
O1^i^—Co1—O1—C6	143.4 (4)	C4—C5—C6—O1	178.6 (5)
N1—Co1—O1—C6	24.7 (4)	C7—C5—C6—O1	−4.6 (8)
N1^i^—Co1—O1—C6	−101.1 (4)	C4—C5—C6—C1	−3.2 (7)
O1—Co1—N1—C7	−18.8 (5)	C7—C5—C6—C1	173.6 (5)
O1^i^—Co1—N1—C7	−166.3 (5)	C8—N1—C7—C5	−171.0 (6)
N1^i^—Co1—N1—C7	90.3 (5)	Co1—N1—C7—C5	5.6 (9)
O1—Co1—N1—C8	157.8 (4)	C6—C5—C7—N1	10.5 (10)
O1^i^—Co1—N1—C8	10.2 (5)	C4—C5—C7—N1	−172.7 (6)
N1^i^—Co1—N1—C8	−93.1 (4)	C7—N1—C8—C9	−124.3 (6)
C6—C1—C2—C3	−1.7 (9)	Co1—N1—C8—C9	59.1 (7)
Br1—C1—C2—C3	−177.1 (4)	N1—C8—C9—C14	−140.6 (6)
C1—C2—C3—C4	−0.9 (9)	N1—C8—C9—C10	41.5 (8)
C1—C2—C3—Br2	177.8 (4)	C14—C9—C10—C11	0.0 (10)
C2—C3—C4—C5	1.3 (9)	C8—C9—C10—C11	178.0 (7)
Br2—C3—C4—C5	−177.4 (4)	C9—C10—C11—C12	−0.2 (13)
C3—C4—C5—C6	0.8 (8)	C10—C11—C12—C13	−0.6 (13)
C3—C4—C5—C7	−176.2 (5)	C10—C11—C12—F1	179.6 (7)
Co1—O1—C6—C1	164.8 (4)	C11—C12—C13—C14	1.4 (11)
Co1—O1—C6—C5	−17.0 (7)	F1—C12—C13—C14	−178.8 (6)
C2—C1—C6—O1	−178.0 (5)	C10—C9—C14—C13	0.9 (9)
Br1—C1—C6—O1	−2.5 (7)	C8—C9—C14—C13	−177.1 (6)
C2—C1—C6—C5	3.7 (8)	C12—C13—C14—C9	−1.5 (9)
Br1—C1—C6—C5	179.2 (4)		

Symmetry code: (i) −*x*+2, *y*, −*z*+2.
